# Health system adaptions to improve care for people living with non-communicable diseases during COVID-19 in low-middle income countries: A scoping review

**DOI:** 10.7189/jogh.13.06006

**Published:** 2023-03-03

**Authors:** Leonard Baatiema, Olutobi A Sanuade, Luke N Allen, Seye Abimbola, Celestin Hategeka, Kwadwo A Koram, Margaret E Kruk

**Affiliations:** 1 Department of Health Policy, Planning and Management, School of Public Health, University of Ghana, Legon, Accra, Ghana; 2 Department of Global Health and Population, Harvard T.H. Chan School of Public Health, Boston, Massachusetts, USA; 3 Department of Population Health Sciences, Spencer Fox Eccles School of Medicine, University of Utah, Salt Lake City, Utah, USA; 4 Department of Clinical Research, Faculty of Infectious and Tropical Diseases London School of Hygiene and Tropical Medicine, London, United Kingdom; 5 School of Public Health, Faculty of Medicine and Health, University of Sydney, Sydney, Australia; 6 Centre for Health Services and Policy Research, School of Population and Public Health, University of British Columbia, Vancouver, British Columbia, Canada; 7 Noguchi Memorial Institute for Medical Research, University of Ghana, Legon, Ghana

## Abstract

**Background:**

During the COVID-19 pandemic, access to health care for people living with non-communicable diseases (NCDs) has been significantly disrupted. Calls have been made to adapt health systems and innovate service delivery models to improve access to care. We identified and summarized the health systems adaptions and interventions implemented to improve NCD care and their potential impact on low- and middle-income countries (LMICs).

**Methods:**

We comprehensively searched Medline/PubMed, Embase, CINAHL, Global Health, PsycINFO, Global Literature on coronavirus disease, and Web of Science for relevant literature published between January 2020 and December 2021. While we targeted articles written in English, we also included papers published in French with abstracts written in English.

**Results:**

After screening 1313 records, we included 14 papers from six countries. We identified four unique health systems adaptations/interventions for restoring, maintaining, and ensuring continuity of care for people living with NCDs: telemedicine or teleconsultation strategies, NCD medicine drop-off points, decentralization of hypertension follow-up services and provision of free medication to peripheral health centers, and diabetic retinopathy screening with a handheld smartphone-based retinal camera. We found that the adaptations/interventions enhanced continuity of NCD care during the pandemic and helped bring health care closer to patients using technology and easing access to medicines and routine visits. Telephonic aftercare services appear to have saved a significant amount of patients’ time and funds. Hypertensive patients recorded better blood pressure controls over the follow-up period.

**Conclusions:**

Although the identified measures and interventions for adapting health systems resulted in potential improvements in access to NCD care and better clinical outcomes, further exploration is needed to establish the feasibility of these adaptations/interventions in different settings given the importance of context in their successful implementation. Insights from such implementation studies are critical for ongoing health systems strengthening efforts to mitigate the impact of COVID-19 and future global health security threats for people living with NCDs.

Over the past three decades, the burden of non-communicable diseases (NCDs) increased significantly in low-and middle-income countries (LMICs), with 2019 reports showing that more than 80% of deaths in LMICs were caused by NCDs. Some studies have projected that NCDs are set to overtake communicable/infectious diseases as the main public health threats in LMICs after 2030 [1]. The main drivers for NCDs (including tobacco use, unhealthy diets, and high alcohol use, among others) have also witnessed a major rise in these settings. Despite this, access to high-quality NCD care remains a major challenge [2]. During the COVID-19 pandemic, the delivery of health services in LMICS has been significantly disrupted, especially among people with pre-existing health conditions [3,4].

To date, LMICs have experienced a high burden of morbidity and mortality from the COVID-19 pandemic [5], as well as major concerns due to rising cases and mortality from COVID-19 among people living with NCD conditions [6]. The NCDs most prominently associated with COVID-19 mortality include diabetes, hypertension, stroke, coronary artery disease, and chronic obstructive pulmonary disease [7,8]. Evidence from Wuhan, China shows that 16.7% of the study population was diagnosed and admitted to hospitals due to COVID-19 reported abnormalities in their heart rhythm, including worsened outcomes from pulmonary hypertension and heart failure, strokes, and transient ischemic attack associated with COVID-19 [9]. Similar findings have also been observed elsewhere [10-12].

People living with NCDs have had difficulties in accessing high-quality health care during the pandemic [13,14], while many COVID-19 patients also live with a NCD. Yet, health care systems in LMICs were less resilient and unprepared to provide uninterrupted health care services for this population. Evidence from the World Health Organization (WHO) shows that over 50% of all countries experienced health care disruptions regarding provision of optimal NCD (hypertension, diabetes, cancer, and cardiovascular diseases), diagnosis, and treatment services [15]. The report also highlighted that access to NCD screening and diagnosis, as well as referral for further treatment and management were compromised because of the COVID-19 pandemic. Disruptions in access to mental health services were also reported [16,17]. The disruptions undoubtedly resulted in increased difficulties of living with NCDs, a rise in COVID-19-related mortality, postponement of clinical appointments and procedures, and difficulty in refilling NCD medications.

Although the pandemic disrupted NCD services globally in both high-income countries (HICs) and LMICs, COVID-19 has exposed the fragility, unresponsiveness, and under-investment of health systems in most LMICs and their inability to optimally respond to public health emergencies and address the health needs of people living with NCDs. Lessons from public health emergencies and from the Ebola outbreak in West Africa [18-20] indicate the need to strengthen primary care facilities. Similar calls have been made during the COVID-19 pandemic [21]. Primary care facilities play a front-line role in epidemic preparedness, prevention, and response [22], especially as they are often a patient’s first point of contact with a health system [23]. Pandemics can increase the burden of primary care facilities, negatively affecting NCD care delivery and access. The COVID-19 pandemic has highlighted the need for adaptations of such primary care services in LMICs with weak health systems [24].

Some countries attempted to adapt and strengthen their health system for optimal delivery of care during the COVID-19 pandemic [14,25-27]. For example, Vietnam established digital support mechanisms for people living with NCDs to improve access to NCD care [28]. Some studies have also highlighted contextual facilitators and barriers to delivery and accessibility of high-quality NCDs care during the pandemic [29,30]. Against this background, two critical questions have arisen – what health system adaptations, interventions, and policies were implemented to improve the delivery and accessibility of health care services for the prevention and management of NCDs during the COVID-19 pandemic, and is there evidence of their impact on NCD care delivery or patient outcomes in LMICs?

We aimed to examine the literature on health system adaptations in response to COVID-19 to improve access for people with underlying health conditions such as NCDs, aiming to explore how to reorient health systems to be more resilient and responsive to the health needs of people with underlying health conditions during public health emergencies.

## METHODS

We followed the Preferred Reporting Items for Systematic Reviews and Meta-Analyses Extension for Scoping Reviews (PRISMA-ScR) checklist [31] to ensure rigor and minimize bias while conducting this review. As our scientific inquiry was broad, we decided on a scoping review over a systematic review to ensure that all relevant literature from different study types and designs were captured given that the literature on COVID-19 is still developing [32].

### Search strategy

We searched Medline/PubMed, Embase, CINAHL, Global Health, PsycINFO, Global Literature on coronavirus disease, and Web of Science using a comprehensive search strategy (**Online Supplementary Document**). The search strategy was developed iteratively by the review team using different MeSH terms and was targeted at data from published primary studies, conference abstracts, unpublished (gray literature) primary studies, and reviews. We included papers published in French and English. The search covered articles published between January 2020 (during the onset of COVID-19 pandemic) and December 2021.

### Eligibility criteria

To ensure broad inclusion, we selected articles that reported on COVID-19, NCDs, people living with NCDs, and health care providers. We included studies reporting on any policy, program, or intervention to improve delivery, access, or utilization of NCD care, or if they contained evidence or information on barriers and facilitators to delivery or accessibility of health care services for the prevention and treatment of NCDs. We excluded studies from high-income countries as we targeted studies that focused on LMICs. **Table 1** summarizes the inclusion and exclusion criteria using the population/intervention/comparison/outcome (PICO) principle.

**Table 1:** Implementation criteria

Eligibility criteria	
**Population**	Studies which reported on people living with NCDs or their NCD patients and their care providers. Health care providers refers to clinic based, community-based, pharmacists, multidisciplinary team, dispensers, health managers, policy makers, unit heads, in-charges and policy managers.
**Intervention**	Health systems strengthening activities tailored toward NCDs care provision and accessibility in primary care settings, in-patient wards, out-patient wards. Health systems strengthening mechanisms, policies and program to improve access to care for people living with NCDs will be included. Policies and interventions targeting health promotion, education, disease prevention, coordination of your care, treatment, rehabilitation and palliative care, disease prevention and screenings, diagnosis and surveillance, counselling or health screenings and medication prescriptions.
**Comparator**	None
**Outcomes**	Results from health care provider practice outcomes were considered
**Study design**	All study designs and gray literature

### Search and selection process

One reviewer (LB) searched for the articles in the selected key electronic health sciences databases (**Online Supplementary Document**). The selection and screening process was conducted in four phases. We first deduplicated the references using the EndNote reference manager (version X9). We then screened the reference titles based on pre-determined inclusion/exclusion criteria and our review question, after which three reviewers (LB, CH, and OAS) independently screened the abstracts. Finally, two reviewers (LB and OAS) independently conducted the full-text screening. We resolved disagreements at any phase through discussion until a consensus was reached. If disagreements persisted, we consulted a third reviewer (CH) for the final inclusion decision. For gray literature reports, conference proceedings or editorials where there may be no abstracts or executive summaries, we screened the full texts and included/excluded them based on the review questions.

### Data charting process

We extracted the studies for synthesis to examine the geographical, policy, contextual and methodological scope of the evidence. The charting process in a scoping review includes sifting and sorting the evidence from eligible articles according to the key thematic components in line with the review’s analytical and conceptual framework [33]: the high-quality health systems strengthening framework for our study. This is to aid the development and refinement of a deductive thematic framework that comprises multiple levels of the health system.

We developed an Excel template to extract all relevant data according to the review questions and thematic domains according to the review’s conceptual/analytical framework. Data on authors, country of study, study aim/objectives, study design, population characteristics, sample, health policies and interventions for NCD care provision, and accessibility were extracted using an excel spreadsheet. Two authors (LB and OAS) independently extracted each of the eligible articles.

### Methodological quality appraisal

A formal assessment of methodological quality is not a typical feature of a scoping review, so we did not include it our scoping review [34].

### Coding, summarizing, and reporting results

We adopted a high-quality health systems analytical/conceptual framework to map, organize, and synthesize the findings, seeking to establish and categorise the evidence based on policies, interventions according to specific NCDs conditions, as well as direct and indirect health system factors. We included an expansive list of literature of all study designs and gray literature; consequently, we did not quality appraisal of eligible studies.

## RESULTS

### Selection of sources of evidence

**Figure 1:**
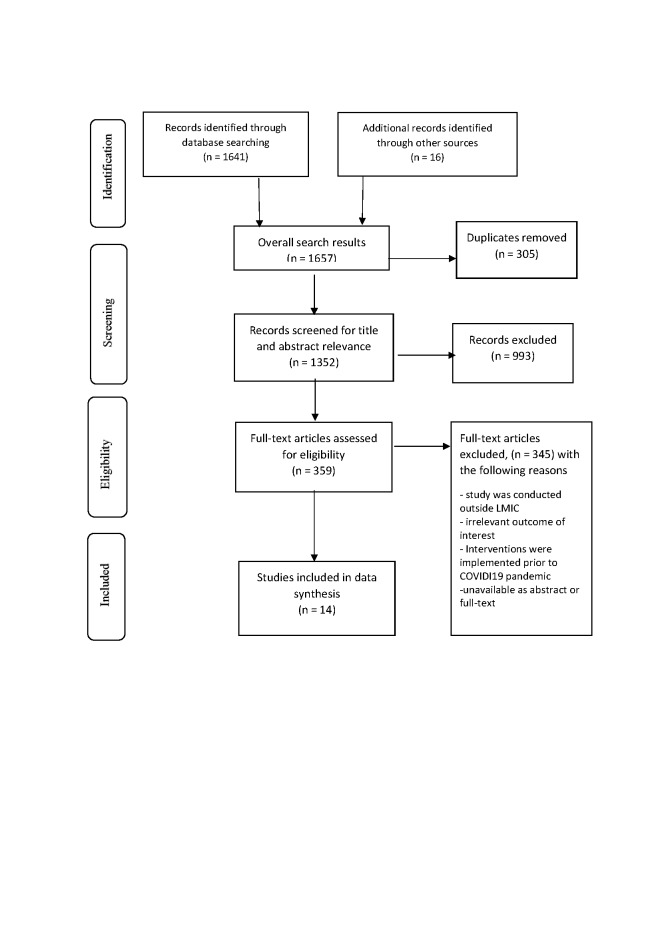
PRISMA 2009 Flow diagram.

We identified 1657 studies from seven electronic databases (APA PsycINFO (n = 58) Health Policy Reference Center (n = 88), CINAHL (n = 31), Embase (n = 455), Global Health (n = 290), MEDLINE (n = 328), Web of Science (n = 291), WHO COVID-19 Global Literature on Corona Virus Disease (n = 100)) and another 16 articles from other sources. We excluded 305 duplicate references. We excluded 993 references during the title and abstract screening and a further 342 during the full-text screening for either being irrelevant to the review aims, scope and study outcomes, or being conducted outside LMICs. We excluded an additional three studies due to a lack of access to the full text. Overall, 14 studies were eligible for the review (**Figure 1**).

### Characteristics of sources of evidence

The included studies were conducted in six LMICs: Peru [35], Brazil [36], India [37-44], Kenya [45], Bosnia and Herzegovina [46], and Mexico [47]. The highest number of studies were from India (n = 9) and Brazil (n = 2), while the rest were from one country study each. Most studies employed a prospective cohort [35,36,39-41,47,48] or cross-sectional design [37,42,46]. Two studies used a qualitative design [44,45] and the rest were clinical audits/review [38] and a field-based report [43]. **Table 2** summarizes the included reports’ characteristics.

**Table 2:** List of eligible studies

No.	Study (year)	Country	Aims/objectives	Study design/report	Participants, age, sample/setting	Type of NCD	Type of intervention
1	Atreya et al. (2020) [37]	India	To assess the changes in the hospital-based practice of palliative care in the COVID-19 pandemic and patient/caregiver's perception about the provision of telehealth services to palliative care patients of a tertiary care cancer hospital of eastern India.	Exploratory survey	50 subjects interviewed telephonically, 54% (n = 27) belonged to the age group of 18-60 y. 56% (n = 28) of the patients were males, while 44% (n = 22) were females.	Cancer	Telemedicine.
2	Naik et al. (2021) [38]	India	experience of immediate adaptation of technology abiding to the latest telemedicine practice guidelines that had aided in maintaining continuity of care to already registered psychiatric patients who were due for their follow-ups during the closure of OPD.	Prospective chart review	1049 patients with psychiatric disorders, mean age of 35, males were 562 (53.6%) and 545 (52%) were above poverty line.	Psychiatric disorders	Telephonic aftercare services, telephonic, follow-up services during the COVID-19 pandemic.
3	Reddy et al. (2021) [39]	India	To determine if decentralization of hypertension follow-up services and free medication to peripheral health centers improved continuity of care and treatment outcomes in hypertensive patients.	Prospective study	Pilot (n = 2315); 6-mo cohort (n = 5527); monthly cohort (n = 1450).	Hypertension	Decentralization of hypertension follow-up services and provision of free medication to peripheral health centers.
4	Shukla et al. (2021) [40]	India	Described problems faced by Parkinson disease patients during lockdown due to lack of health care and medication procurement and addressed such problems using telemedicine via virtual OPD setup	Prospective study	Not reported.	Parkinson disease	Tele-medicine follow-up of patients.
5	Sidana et al. (2020) [41]	India	This study evaluated the need, knowledge, accessibility, and effectiveness of the telemedicine services in community outreach clinic	Prospective study	Mean age = 43.9, male (48%); female (52%), sample size (n = 78).	Mental health	Telemedicine services for the delivery of mental health services.
6	Ullas et al. (2021) [42]	India	to gauge the perception and adoption rates of telemedicine among patients with NCD as opposed to in-person consultations in a quaternary care center in South India.	Cross-sectional study	220 responses.	NCDs	Telemedicine.
7	Philip et al. (2022) [43]	India	MH tele-training toward capacity building of PCDs during the COVID 19 pandemic.	cross-sectional survey	114 PCDs enrolled for the training. Their average age was 32 y, and they reported a mean experience of four years.	Mental health	Digital introductory MH training short-term synchronous training.
8	Nair et al. (2021) [44]	India	Explore provider perspectives in providing mental health services to communities to understand the impact on services and adaptations during the COVID 19 pandemic.	qualitative study	In-depth interviews with 10 service providers; FGD with 4 service providers.	Mental health	Telephone/ videoconferencing.
9	Queiroz et al. (2020) [36]	Brazil	To evaluate diabetic retinopathy screening with a portable handheld smartphone-based retinal camera and telemedicine in an urban primary health care setting.	Prospective study	627 adult individuals with T2DM.	Type 2 diabetes mellitus (T2DM)	Diabetic retinopathy screening strategy with a handheld device and telemedicine.
10	Salum et al. (2020) [48]	Brazil	To test the feasibility of implementing intensive telehealth case management system to fight the COVID-19 pandemic in a community psychosocial center in Brazil.	Prospective study	Mean age = 38.8 (SD = 13.6) years. 61% were males. Sample comprised 154 patients.	Psychosis, intellectual disability, other mental health conditions	Telehealth case management.
11	Silva-Tinoco et al. (2021) [47]	Mexico	This study evaluated the conversion of an outpatient diabetes primary care center from a face-to-face care modality to a telemedicine care service by telephone.	Prospective study	Mean age = 54.1 y, male = 33.9%, female = 66.1%, sample = 192.	Diabetes	Telemedicine care service by telephone.
12	Angulo et al. (2021) [35]	Peru	To evaluate the utility of telemedicine in the adherence of the treatment of patients with DM1 in Peru, during COVID-19.	Prospective study	25 patients with DM1. 17 girls and 8 boys, an average age of 12 y, an average debut age of 9 y, an average time of illness of 4 y and the average insulin dose was 1UI/kg/d	Type 1 diabetes	Teleconsultation strategy zoom platform, messages by WhatsApp weekly with parental consent.
13	Pajević et al. (2020) [46]	Bosnia and Herzegovina	To explore the organization of psychiatric services in BH to meet mental health needs of BH citizens during the particularly restrictive measures caused by COVID-19 pandemic.	Cross-sectional survey	38 study participants	Mental health	Telepsychiatry.
14	Kiragu et al. (2021) [45]	Kenya	To describe the challenges faced in accessing NCD medicines in Kenya during the pandemic, through a descriptive narrative informed by key stakeholders engaged in NCD service delivery and decision-making.	A descriptive narrative approach	County officials, service providers and Ministry of Health officials	diabetes and hypertension	A Medicines Delivery Initiative – NCD medicines drop-off points in Bungoma county.

NCD – non-communicable disease, MH – mental health, PD – primary health doctors, OPD – outpatient department, BH – Bosnia and Herzegovina, DM1 – diabetes mellitus 1, SD – standard deviation, y – years, mo – months

### Synthesis of results

We synthesized the different adaptations, interventions, and repurposing of health systems to improve access to care for people living with NCDs. We also report on interventions based on the specific NCD conditions targeted.

#### Health systems adaptations to improve access to NCDs care during the COVID-19 pandemic

**Figure 2:**
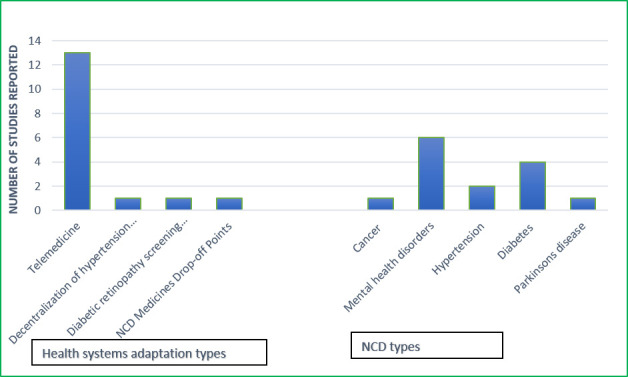
Health systems adaptations and NCD types according to the number of reported studies.

We identified four different health systems adaptations and reconfiguration to improve access to NCDs: telemedicine or teleconsultation strategies [35-38,40,41,43,44,46-48], NCD medicines drop-off points [45], decentralization of hypertension follow-up services and provision of free medication to peripheral health centers [39], and diabetic retinopathy (DR) screening with a handheld smartphone-based retinal camera [36]. Telemedicine was the most introduced intervention among the four. It was conducted through telephone calls [44,47], WhatsApp [35], and video calls and conferencing [44]. Among the telemedicine strategies, telepsychiatry was widely used to provide care to people living with mental health disorders [38,41,43,44,46,48]. One study reported the use of telemedicine to train health care providers to provide mental health care during the COVID-19 pandemic. **Figure 2** presents the health systems adaptations and NCD types according to the number of reported studies.

#### Health systems adaptations and NCD types

The 14 identified studies reported on different populations living with NCDs. Six studies reported on mental health disorders [38,41,43,44,46,48], four focused on people living with diabetes [35,36,45,47], and the rest were related to cancer [37], hypertension [39,45], and Parkinson disease [40]. For diabetes care, most of the adaptations were telemedicine strategies [35,36,47], while others comprised NCD medicines drop-off points [45] and DR screening with a handheld device [36]. Telepsychiatry was also used to address the needs of people with psychiatry and mental health disorders [38,41,43,44,46,48]. Due to the COVID-19 pandemic, one study reported on the training of health care providers synchronously on mental health care to support virtual care for people with mental health conditions held [43]. People with Parkinson disease were also targeted as part of health systems improvement measures during the COVID-19 pandemic. One study reported on the use of telemedicine to follow up on patients with Parkinson disease [40]. Two different interventions focused on providing care for people with hypertension namely the use of NCD medicine drop-off points [45], and decentralization of hypertension follow-up services from major health care delivery points to peripheral health centers [39]. One study reported on health systems adaptations to improve cancer care. This was through a telemedicine-driven intervention to provide health care support for cancer survivors [37]. **Table 3** outlines the distribution of studies according to health systems adaptation types and NCDs.

**Table 3:** Health systems adaptations/NCD types and the associated number of reported studies

Health system adaptation type	NCD type	Number of NCDs
Telemedicine	Mental health, cancer, hypertension, diabetes, Parkinson disease	5
Decentralization of hypertension follow-up services/provision of free medication to peripheral health centers	Hypertension	1
Diabetic retinopathy screening strategy with a handheld device	Diabetes	1
A Medicines Delivery Initiative – NCD medicines drop-off points	Hypertension and diabetes	1

NCD – non-communicable disease

#### Impact of service delivery adaptations and interventions for NCDs care during COVID-19

Although we identified four health systems adaptations/interventions to improve NCD care, three interventions had reported results of potential impact (**Table 3**).

##### Telemedicine

Telemedicine was employed in improving access to care among people living with diabetes, cancer, and Parkinson disease in India, Brazil, Mexico, Peru, Bosnia and Herzegovina, and Kenya. The findings revealed variations in the potential impact of telemedicine on NCD care. In Peru, the use of teleconsultation during the COVID-19 pandemic for diabetes care was associated with better treatment adherence with 90% glycaemic control and greater commitment to the diabetes management [35]. Similar results were reported in Mexico where almost all participants (95%) considered the telemedicine strategy as a useful approach to managing their diabetes condition [47].

Besides diabetes, telemedicine was deployed to improve access to mental health care. The review reported that telepsychiatry was widely used in improving access to the mental health care before and after teleconsultations were introduced during the COVID-19 pandemic [38,41,46,48], training of health care providers on mental health care [43], and improved efficiency of service delivery and service coverage [44]. Similar telepsychiatry care services were associated with improved accessibility of services by mental health patients [41,44] and improved outreach services to mental health patients who required routine follow-up care [38] before and after teleconsultations were introduced during the COVID-19 pandemic.

Telemedicine was also used to improve access to cancer care during the COVID-19 pandemic. A survey was conducted in India to evaluate the changes in the hospital-based practice of palliative care following the pandemic and to explore the views of patients and their carers about the use of telehealth services to provide palliative care. The researchers reported that telemedicine was associated with a decline in outpatient clinic footfalls by 51%, and a decline in inpatient admissions by 44%. Regarding their perceptions of the telehealth services for palliative care, about 82% of patients/caregivers gave positive feedback about telemedicine and 64% of patients and caregivers felt assured about the available support system [37]. Consequently, 76% of the participants agreed they would consider using teleconsultation services in the future.

People living with Parkinson disease were also reported to have benefited from a telemedicine-adapted health service delivery strategy. Findings from India noted that the use of telemedicine services using video call sessions facilitated contact with patients during the nationwide lockdown, encouraged prompt response to patient needs and helped to alleviate the fear of COVID-19 [40]. The telemedicine service also aided in maintaining the functionality of health care services to respond to the needs of people living with Parkinson disease without any additional cost.

##### Diabetic retinopathy screening with a portable handheld smartphone-based retinal camera and telemedicine

In a prospective study conducted in Brazil to evaluate DR screening with a portable handheld smartphone-based retinal camera and telemedicine in an urban primary care setting during the COVID-19 pandemic, the findings observed that referral decision was possible in 81.2% patients [36]. The study also showed that most patients had absent or non-referable DR and the daily rate of patients whose images allowed clinical decision was maintained above 80%.

##### Decentralization of hypertension follow-up services

A key adaptation we identified is the strategy of decentralising hypertension care services from major referral points to peripheral areas in India. This intervention comprised the relocation of hypertension care services closer to the community and the provision of free medication to peripheral health centers [39]. This study was carried out to evaluate if decentralising follow-up services for hypertension as well as providing free medication to peripheral health centers improved continuity of care and treatment outcomes and maintained service continuity during the COVID-19 pandemic. This prospective study showed that, in the decentralized facilities, hypertensive patients recorded much higher rates of follow-up as well as increased blood pressure control than in the non-decentralized facilities. The blood pressure (BP) control rates had similar patterns to the follow-up rates and were higher in the intervention groups than in the non-intervention groups. Among all newly registered patients at the India Hypertension Control Initiative (IHCI patients), a higher percentage of those accessing decentralized facilities/districts had their BP under control – 74% for the 2019 pilot cohort (which is close to the national target of 80%) and 61% for the April 2020 cohort – compared with patients from non-decentralized facilities/districts (65% for the 2019 pilot cohort and 32% for the April 2020 cohort) (**Table 4**).

**Table 4:** Summary of evidence on impact of health systems adaptations and intervention models to improve NCD care during COVID-19 pandemic

NCD type	NCDs service delivery adaptations and intervention	Potential impact	Study (year)
Diabetes	Telemedicine, teleconsultation strategy	Teleconsultation for diabetes care resulted in better treatment adherence, a 90% glycaemic control, greater commitment to diabetes management	Angulo et al. (2021) [35]
	Telemedicine care service by telephone	A total of 1118 consultations were made by telephone and follow-up was subsequently continued in 192 patients with type 2 diabetes. Different professionals from different health areas participated in the telemedicine care service delivery. Nearly all the patients (95%) of the patients considered telemedicine strategy useful for the management of their disease.	Silva-Tinoco et al. (2021) [47]
Psychiatry and mental health disorders	Telephonic aftercare services telephonic follow-up services	Telephonic aftercare services save a lot of time and money for patients. Telephonic consultations are not only feasible, but also provide useful means to reach out to patients, who particularly require routine follow-up consultations.	Naik et al. (2021) [38]
	Telehealth case management	Telehealth case management reduced risks of psychiatric instability from stress related to COVID-19.	Salum et al. (2020) [48]
	Telemedicine services	Increased feasibility, acceptability, and accessibility of mental health service delivery in community outreach clinics	Sidana et al. (2020) [41]
	Digital introductory MH training – short-term synchronous training	37% improvements in knowledge scores of the participants on case-based scenarios on providing mental health care services to clients	Philip et al. (2022) [43]
	Telemedicine services	Telemedicine enabled expansion of service and clientele as well as efficiency, but there were issues of casualization of therapy and poor privacy.	Nair et al. (2021) [44]
Hypertension	Decentralization of hypertension, services and provision of free medication to peripheral health centers	In the decentralized facilities, hypertensive patients recorded a much higher rates of follow-up and as well controlled their blood pressure than in the non-decentralized facilities. The BP control rates had similar patterns to the follow-up rates and were higher in the intervention groups. Differences in follow-up and hypertension control rates were statistically significant (*P* < 0.001) for the 2020 six-month cohort. For the decentralized groups, the follow-up rate was 86% in the 2019 pilot cohort and 78% in the March 2020 cohort, a 9% difference. for the non-intervention groups, the follow-up rate was 74% in the 2019 pilot cohort and 36% in the March 2020 cohort	Reddy et al. (2021) [39]
Cancer	Telemedicine	Telemedicine resulted in a decline in outpatient clinic footfalls by 51%, inpatient admissions reduced by 44%, about 82% of patient/caregivers gave positive feedback about telemedicine and that 64% of the patients and caregivers felt assured about available support system	Atreya et al. (2020) [37]
Parkinson disease	Tele-medicine follow-up of patients	Facilitated contact with patients during the nation-wide lockdown, encouraged prompt response to patient needs and helped to alleviate the fear of COVID-19; aided in maintaining functionality of health care services to respond to the needs of people living with Parkinson disease without any additional cost.	Shukla et al. (2021) [40]
NCDs	Telemedicine	In-person consultations decreased by 1.9 ± 4.47 visits per year, in 2020 vs 2019. Consultation times had significantly decreased (OR = 6.43, 95% CI = 1.7-24.08, *P* = 0.006). Difficulty in obtaining in-person appointments, along with the reduced physical examination during consultations	Ullas et al. (2021) [42]

NCD – non-communicable disease, MH – mental health, OR – odds ratio, CI – confidence interval

Differences in follow-up and hypertension control rates were statistically significant (*P* < 0.001) for the 2020 six-month cohort, and for the 2019 pilot the sample sizes considerably rendered the difference in rates. Follow-up rates were also stable and maintained among patients in the decentralized facilities while those in non-decentralized facilities recorded a lower follow-up [39]. For example, for the decentralized groups, the follow-up rate was 86% in the 2019 pilot cohort and 78% in the March 2020 cohort, a 9% difference. In contrast, for the non-intervention groups, the follow-up rate was 74% in the 2019 pilot cohort and 36% in the March 2020 cohort, a decrease of 51%. Overall, continuity of care was maintained during the COVID-19 pandemic because of decentralisation of hypertension care services. A key characteristic of the strategy of hypertension care services’ decentralisation was the delivery of free medicines and follow-up services to most peripheral primary care facilities.

## DISCUSSION

The onset of the COVID-19 pandemic disrupted access to preventive and treatment services for NCDs. We identified and synthesized evidence on the health systems and policy adaptations across LMICs to improve and minimise disruptions in access to care among people living with NCDs.

### Overview of findings

We synthesized 14 peer-reviewed journal articles, reports, and abstracts to provide an analytical overview of the health systems adaptations and changes to address the barriers faced by people living with NCDs in accessing care. We identified four different health policy and system adaptations: 1) telemedicine or teleconsultation strategies, 2) NCD medicines drop-off points 3) decentralization of hypertension follow-up services and provision of free medication to peripheral health centers, and 4) DR screening with a handheld smartphone-based retinal camera. However, the use of telemedicine or teleconsultation emerged as the widely implemented health policy adaption during the COVID-19 pandemic in most of the countries where these studies were reported from. The telemedicine services were provided in the form of telephone consultations, video consultations, or through WhatsApp. The predominant use of teleconsultation is not surprising, as the uptake of teleconsultation services increased over the past few years in LMICs [49]. We highlighted the need for policy actions to support the implementation of telemedicine interventions in LMICs, specifically addressing barriers that impede efforts to improve access to teleconsultation care for NCDs and other conditions.

### Comparison with previous literature

Recent research has highlighted the rising burden of NCDs in LMICs and the need to improve care [2,50]. The COVID-19 pandemic has pointed to the need for improving access to NCDs preventive and treatment services as new evidence linked COVID-19 mortality, complications, and morbidity with people living with NCDs [9,51]. During the COVID-19 pandemic, some of the reported health system adaptions to improve NCDs care included the use of teleconsultation services and the decentralization of NCD care. We identified telemedicine as the most common and widely applied health systems adaption in LMICs to improve NCD care, most especially diabetes [35], mental health disorders [41,46,48], and hypertension care [39]. This finding re-emphasizes increasing policy interest in the use of telemedicine to improve access to NCD care and other health conditions, particularly during the COVID-19 pandemic [52-54]. The pandemic sparked an interest in the use of telemedicine following state policy responses such as lockdowns, closure of public facilities, and disruption in routine care in most health facilities [55,56]. Consistent with our findings, the use of telemedicine was found to be favorable among people living with NCDs, with evidence of potential impact [52,57]. For example, a recent scoping review showed that the use of telemedicine resulted in a significant reduction in the blood pressure of participants because of telemedicine intervention [57]. Similarly, in Thailand, delivery of health services was decentralised and telemedicine uptake was promoted to ensure continuity of care with minimal disruption as a result of the pandemic [58].

Decentralising NCDs care is increasingly gaining traction [59-61], especially in response to the treatment and access gaps associated with the provision of optimal NCDs care during the pandemic. Consistent with the findings of this review, decentralization of NCDs care has also been reported in different contexts. We observed that, following the pandemic and the associated disruptions in care, some health facilities adopted a policy of decentralising NCD care to ensure people living with NCD were still able to refill their medications and other health services at their doorstep, though they implemented the policy in different ways. For example, we found that access to NCDs care was decentralised in India where screening and diagnosis, monitoring of NCDs, and dispensing or medicine refills were shifted from the secondary or higher level of care to lower levels such as the subcenters, the peripheral health facilities [39]. Unsurprisingly, Reddy et al. [39] reported that as a result, hypertensive patients in decentralised facilities recorded much higher rates of follow-up and blood pressure control than in the non-decentralized facilities. A recent study in Eswatini also reported that the decentralisation of the management of diabetes and hypertension care to nurses in community clinics yielded better health outcomes, evident in a significant reduction in mean BP among hypertensive, and a non-significant reduction in fasting blood glucose among diabetic patients [60]. Although not a popular health system and health policy adaptation to improve NCD care, this evidence highlights its potential in decentralising NCD care post-COVID-19. Consequently, calls to decentralise NCDs care have been made to ensure the continuity of the uninterrupted care [62-64].

Access to regular retinal screening among people living with diabetes is recommended for early detection and timely treatment of diabetic retinopathy (DR). However, access to such screening services remains a major challenge due to limited ophthalmologists or screening using retinal color photography. To ensure uninterrupted access to retinal screening, our review identified a study in Brazil where a portable handheld smartphone-based retinal camera was used to screen for DR [36]. Although the convention is for an ophthalmologist to undertake fundus examination or conduct a retinal color photography using conventional mydriatic or non-mydriatic fundus cameras by optometrists or trained eye technicians [65], we found that referral decision was possible in 81.2% patients in the Brazilian study. The study also showed that most patients had absent or non-referable DR using the portable handheld smartphone-based retinal camera for screening of DR and that the daily rate of patients whose images allowed clinical decision was maintained above 80%. These findings similar to those of previous works [66,67]. The findings from this intervention adds to the growing body of evidence about emerging strategies of improving access to diabetes retinopathy screening. This is important because DR has emerged as a leading case of new blindness of adults 20-70 years and about 95% of the DR cases can be averted through early screening and referring for treatment [66]. Although DR screening approaches are developing and there is scope for new and emerging tools and approaches, the use of the handheld. Consequently, the portable handheld smartphone-based retinal camera for screening of DR during the COVID-19 pandemic ensured continuity of services to prevent further cases of DR.

Geographical access to NCD care remains integral in current discourses around reducing the growing burden of NCDs. Past works have observed that people living with NCDs face several barriers to accessing better NCD care [68-71]. Findings from the WHO surveys highlighted geographical access to NCDs care as a key undermining factor to continuity of care and called for action to address this [4,15,72]. A key adaptation we identified was the use of NCD medicine drop-off points to ensure continuity of NCDs care during the COVID-19 pandemic [45]. This study showed that, following the COVID-19 pandemic, the Kenyan government adapted the Medicines Delivery Initiative dubbed as the NCD medicines drop-off points where essential medicines for NCDs (hypertension and diabetes) were delivered to specific pick-up points in the community with an arrangement to supply every three months, with communities playing a role in scheduling, pick up points, or locations for the supplies. A similar adaption was reported in Thailand, where medicines pick up points were established as part of government innovations to improve access to NCDs care [58]. Although out-patient services were observed to have declined due to the pandemic, the community delivery of medicines program potentially led to better control of hypertension diabetes as there was minimal interruptions of NCDs care.

### Implications for policy, research, and public health practice

We outlined different health systems adaptations made to improve uninterrupted access to care for people living with NCDs during COVID-19 in LMICs. Telemedicine was the most reported health systems adaptation to improve access to NCD care, with potentially significant impact. Healthcare managers and policymakers interested in health systems adaptations to improve care during future pandemics of outbreaks may consider investing in telemedicine services especially because it is more impactful for NCDs care such as mental health disorders, hypertension, and diabetes care.

There is evidence that the use of telemedicine or teleconsultation services presents a potent and promising service delivery intervention to improve access to NCD prevention, treatment and management services in future public health pandemics. This review demonstrated that this service was implemented to improve diabetes care, cancer and hypertension care. One study also reported that the intervention was employed to remotely train health care providers to improve their knowledge of mental health care delivery. This information emphasized the need to explore avenues to invest more in telemedicine services to improve care. Several studies reported diabetes and mental health disorders as the predominant NCDs that these health systems adaptations sought to address. However, the scalability of the health systems measures established and implemented to improve access to care among people living with other NCDs conditions such as stroke and heart disease remains unclear. Implementation research to unpack the contextual enablers and barriers underpinning the successful implementation of these interventions is needed. Telemedicine is already being promoted as a pathway to close the treatment and access gap for optimal NCD care, so it is unsurprising that we identified it as the most predominant health system adaptation intervention to improve access to NCD care during the COVID-19 pandemic. Various state governments, ministries of health, and partners should invest more in telemedicine services to strengthen health systems. However, there is also little information on how to scale up these interventions in resource-limited settings in ways that improve health system function. Future research must explore the implementation strategies and outcomes of using telemedicine in LMICs. For example, given that telemedicine was the widely used intervention, immediate research steps may be taken to clearly map out the contextual factors driving the use of telemedicine for NCD care in this setting.

Decentralisation of NCD treatment services to improve access to care and address the long-standing treatment gap was also notable although not widespread. Past works in Vietnam, Brazil, other countries Ethiopia, Eswatini [60], Kenya [73], Uganda [74] and Rwanda [61], where community health care workers (CHWs) have been tasked with NCD care, reported on the decentralisation of NCD care in Africa with demonstrable potential impact on clinical outcomes. Unsurprisingly, we noted this was reported as an intervention to improve high blood pressure care during the pandemic in India. This is an innovative approach that should be given policy attention. To date, most NCD treatment services are highly centralised and most primary care facilities especially at the local health service levels do not provide comprehensive care for NCDs. Most services are provided primarily in urban tertiary facilities thus limiting access to optimal NCDs for most patients. We demonstrated the utility of decentralising NCDs care to the doorstep of the people in need of such services. Post COVID-19, such low-cost, health systems adaptation interventions should be expanded to improve NCD screening and treatment, especially drug refill in primary care facilities. This will minimise the congestion in major referral hospitals in LMIC and reduce or eliminate out-of-pocket payments often characterized by a referral from primary care to tertiary health facilities.

### Limitations

This review has several limitations. First, we performed a narrative synthesis of the evidence identified from the eligible studies which were drawn from all types of studies, including gray literature. Hence, we present evidence of the potential impact of the different interventions and health systems adaptations to improve NCD care during the COVID-19 pandemic. Most of the included studies used relatively weak designs and many were not able to establish causality. The primary aim of this scoping review was to identify adaptations rather than critique their contribution. The findings should thus be interpreted with this in mind. Future attempts to measure the actual impact of these interventions may consider experimental study designs, and more importantly, study designs that allow detailed exploration of why and under what circumstances the adaptions worked (or not) to improve access to high-quality NCD care. Second, although we adopted a broader and less restrictive approach in conducting the research, given that the search was restricted to the English language, it is possible we overlooked some important, eligible articles. Also, few of the studies addressed how to incorporate this within regular health systems and how this work when services are routinely operating. It is likely we missed numerous health service modifications that have not yet been published. We mitigated this by looking to gray literature, yet notably the interventions in the review do not represent all potential adaptations.

## CONCLUSIONS

We found that different health systems and policy adaptations have been implemented to improve access to care across different LMIC contexts and demonstrated the potential impact of some of them. Lessons from countries where these were successfully implemented should be shared to guide implementation in other, similar contexts. Further research is needed to comprehensively evaluate the impact of such interventions on health, user confidence, and overall health system performance. Insights from such research endeavors are critical for ongoing health systems strengthening efforts to mitigate the impact of COVID-19 and future global health security threats for people living with NCDs. Future research should also examine the contextual barriers and enablers toward the implementation of such measures. The use of telemedicine in the prevention and treatment of infectious diseases is expanding, with evidence of the potential impact on optimizing clinical outcomes and improving access to care. Our findings are similar for NCD prevention and treatment during COVID-19, revisiting the debate on the potential of telemedicine and other innovations to improve access to care and address treatment gaps for NCDs. However, it is crucial that well-designed implementation studies are conducted to inform adoption and scale up in different settings.

## Additional material


Online Supplementary Document
