# Supplementary material for: Health system adaptions to improve care for people living with non-communicable diseases during COVID-19 in low-middle income countries: A scoping review
Source: J Glob Health. 2023 Mar 3;13:06006. doi: 10.7189/jogh.13.06006 (PMC9980283; doi:10.7189/jogh.13.06006)
Supplement: Online Supplementary Document [file jogh-13-06006-s001.pdf]

Supplementary file 1: Medline Search Strategy

Search	Query
**1**	Coronavirus or COVID-19 or SARS CoV-2 or Coronavirus disease 2019 or 2019-nCoV and 2019-nCoV OR Acute respiratory disease or Coronavirus Pandemic (COVID-19) OR pandemic or epidemic or disease outbreaks or SARS or severe acute respiratory syndrome or coronavirus or SARSCoV-2 or COVID or Novel Coronavirus
**2**	Primary Care Nursing or primary care or Primary Health Care or primary medical care or primary healthcare or family practice or general practice or family physician or family doctor or community health services or primary care or primary healthcare or primary medical care or general practice or GPs or family physician or Hospital or clinics or community health service or Primary care OR therapy OR PHC primary health care surveillance or hospital settings or district hospitals or provincial hospital or general hospitals
**3**	Alzheimer or Alcohol or dietary choices or Anxiety or Asthma or arthritis or cancer or cancers or Cardiovascular diseases cardiovascular or cardio-vascular or cerebrovascular or cerebrovascular or cerebrovascular diseases or Chronic Conditions or Chronic Lung Diseases or Chronic NCDs or Chronic Non-Communicable Diseases or chronic obstructive or chronic obstructive pulmonary disease or Chronic Respiratory Diseases or Chronic Stress or Chronicity or coronary or Coronary heart diseases or Coronary artery disease or Congenital heart defects or Dementia or Depression or Diabetes or Diabetes Mellitus or Diabetes Mellitus or Type 2 or Emotional Stress or Heart Attacks or heart diseases or Heart Failure or High Blood Pressure or Huntington or Hypertension or ischaemia or ischemia or ischaemic or Ischaemic Heart Disease or ischemia or ischemic or Kidney Disease or lung diseases or malignant or Mental Health or myocardial infarct or Myocardial Infarction or NCDs or Neurodegenerative Disorders or Non-Communicable Diseases or Obesity or Stress or Overweight Parkinson or Pulmonary edema or lung injury or Congenital heart disease or risk or Sickle Cell or sickle cell disease or Stroke or tumor or tumour or type 2 or type ii or vascular or vascular diseases or vascular event or oncologic or operative or surgical or psychiatric or rheumatologic or immunologic or neurologic or gastrointestinal, ophthalmologic and endocrine or neurologic disorders or schizophrenia or bipolar disorder
**4**	Developing Countries OR Africa or Caribbean or West Indies or South America or Latin America or Central America or Benin or Burkina Faso or Burkina Fasso or Upper Volta or Burundi or Uganda or Central African Republic or Chad or Comoros or Comoro Islands or Comores or Mayotte or Congo or Democratic Republic of Congo or Republic of Zaire or Zaire or Eritrea or Ethiopia or Gambia or The Gambia or Guinea or Guinea Bissau or Liberia or Madagascar or Malagasy Republic or Malawi or Nyasaland or Mali or Mozambique or Niger or Rwanda or Ruanda or Sierra Leone or South Sudan or Tanzania or Togo or Togolese or Togolese Republic or Uganda or Zimbabwe or Cape Verde or Cabo Verde or Cameroon or Cameron or Camerons or Cote d'Ivoire or Ivory Coast or Ghana or Guiana or Guyana or Gold coast or Kenya or Lesotho or Basutoland or Mauritania or Nigeria or Sao Tome or Principe or Senegal or Swaziland or Zambia or Zimbabwe or Rhodesia or Cambodia or Khmer Republic or Kampuchea or Republic of Korea or North Korea or Korea or Kiribati or Laos or Lao or Lao Democratic Peoples Republic or Lao PDR or Federated States of Micronesia or Micronesia or Papua New Guinea or Philippines or Philippines or Samoa or Samoan Islands or Solomon Islands or Vanuatu or New Hebrides or Vietnam or Vietnam or Afghanistan or Somalia or Djibouti or French Somaliland or Egypt or United Arab Republic or Morocco or Ifni or Sudan or Syria or

	Syrian Arab Republic or West Bank and Gaza or Palestine or Yemen or Republic of Yemen or Bolivia or Guatemala or Guyana or Guiana or Honduras or Nicaragua or Haiti or El Salvador or Nepal or Bangladesh or Bhutan or India or Indonesia or Myanmar or Burma or Myanma or Pakistan or Sri Lanka or Timor leste or East Timor or East Timur or Armenia or Armenian or Georgia or Georgian Republic or Georgia Republic or Kosovo or Kyrgyz Republic or Kyrgyzstan or Kirghizia or Kyrgyz Republic or Kirghiz or Kirgizstan or Moldova or Moldovia or Tajikistan or Tadzhikistan or Tadjikistan or Tadzhik or Ukraine or Uzbekistan or Uzbek or Middle East or developing or less developed or under developed or underdeveloped or middle income or low income or underserved or deprived or poor or middle income or low income or LMICS or third world or Transitional countr*
**5**	1 AND 2 AND 3
**6**	4 AND 5
**Limiters**	2020-2021<br>Full Text<br>English Language<br>Humans
